# Supplementary material for: Logical gates in ensembles of proteinoid microspheres
Source: PLoS One. 2023 Sep 18;18(9):e0289433. doi: 10.1371/journal.pone.0289433 (PMC10506713; doi:10.1371/journal.pone.0289433)
Supplement: Attachment — Submitted filename: Response to Reviewers.pdf [file pone.0289433.s013.pdf]

Response to Reviewer Comments on "Logical Gates in Ensembles of Protenoid Microspheres"

Reviewer 1

All figures must be improved for better visibility (increase the letters/numbers, make lines thicker and points enlarged. In other words, please make all parts of the figures better visible. Do not use light colors like yellow, for example.

We have improved the figures 1, 2, 3, 7, and 11 as per your suggestion. We have increased the font size, contrast, and resolution of the figures to make them more visible and clear.

Do the authors really mean an oscilloscope in Figure 3? Oscilloscopes were used from 1930s till 1980s. They are old instruments. Normally, they are not used anymore being substituted with modern computerized electronics. Potentially, they might be used now, but it looks strange.

We have updated the figure and corresponding text to reflect the use of a potentiostat for the chronoamperometry measurements.

Do not use the reference to the German version of a paper published in Angew. Chem. Please change it to the English version, which will be more convenient for international readers. If needed, they can find the German version.

We have changed the citation to the English version of the paper “Molecules That Make Decisions” by Alberto Credi, which was published in Angewandte Chemie International Edition in 2007

The technical details on the electrochemical experiments should be provided. They include:
a) the kind of the electrochemical instrument (name, company, etc.)
The Zimmer Peacock potentiostat Anapot EIS ZP1000080 was utilised for conducting chronoamperometry measurements.
b) the reference electrode (e.g., Ag/AgCl or calomel, etc.)
A two-electrode setup was used for the electrochemical measurements, with iridium-coated stainless steel sub-dermal needle electrodes (Spes Medica S.r.l., Italy) serving as the working and counter/reference electrodes.
c) the initial and final potentials used in the chronoamperometric measurements
The potentiostat applied a constant dc potential (Edc) and measured the current response at 0.1 s intervals for 25,000 s. For the results shown, Edc was 0.01 V, with no initial equilibration period.
d) the composition of the electrolyte solution, its pH, etc.

The electrodes were positioned approximately 10 mm apart in a protenoid solution containing 10 mg/100 ml protenoid in water as the supporting electrolyte.
e) the kind of the working and counter electrodes (e.g., gold or glassy carbon, etc.)
See also b)

Reviewer 2
In the introduction, it would be very beneficial to discuss briefly previous accounts of electrochemical logic gases, e.g. based on those papers:
1. AMATORE, C.; BROWN, A. R.; THOUIN, L.; WARKOCZ, J.-S., Mimicking neuronal synaptic behaviour: Processing of information with 'AND' or 'OR' Boolean logic via paired-band microelectrode assemblies. C. R. Acad. Sci. Paris 1998, 509-515.
2. Amatore, C.; Thouin, L.; Warkocz, J.-S., Artificial Neurons with Logical Properties Based on Paired-Band Microelectrode Assemblies. Chem. Eur. J. 1999, 5, 456-465.

We have added a paragraph to the paper based on the following papers:
1. AMATORE, C.; BROWN, A. R.; THOUIN, L.; WARKOCZ, J.-S., Mimicking neuronal synaptic behaviour: Processing of information with 'AND' or 'OR' Boolean logic via paired-band microelectrode assemblies. C. R. Acad. Sci. Paris 1998, 509-515.
2. Amatore, C.; Thouin, L.; Warkocz, J.-S., Artificial Neurons with Logical Properties Based on Paired-Band Microelectrode Assemblies. Chem. Eur. J. 1999, 5, 456-465.
"The field of electrochemical logic gates has seen significant research activity. Amatore and colleagues were among the early pioneers in this area, showcasing the capabilities of paired-band microelectrode assemblies. These assemblies successfully imitate the behaviour of neuronal synapses and are capable of performing Boolean logic operations. Amatore et al. demonstrated that artificial neurons utilising coupled double-band electrodes have the capability to operate as AND and OR logic gates [1],[2]. This is achieved by leveraging the distinctive diffusional cross-talk effects in close proximity to the electrodes. A detailed investigation was conducted on the time responses and theoretical features of these electrochemical logic gates. Expanding upon the encouraging progress made with paired microband electrodes as electrochemical logic gates, our current research focuses on the development of protenoid microsphere-based logic gates. Protenoid microspheres serve as a biomolecular platform that integrates the propagation, transmission, and detection of signals, similar to how natural neurons function."

Some other references to electrochemica logic systems may be also added for completness of the picture.

We have added literature from the following papers to our paper:

Zhang, L., Wang, H.X., Li, S. and Liu, M., 2020. Supramolecular chiroptical switches. *Chemical Society Reviews*, *49*(24), pp.9095-9120.
Willner, I., Willner, B. and Katz, E., 2007. Biomolecule–nanoparticle hybrid systems for bioelectronic applications. *Bioelectrochemistry*, *70*(1), pp.2-11.
Valov, I., Waser, R., Jameson, J.R. and Kozicki, M.N., 2011. Electrochemical metallization memories—fundamentals, applications, prospects. *Nanotechnology*, *22*(25), p.254003.

Experimental part misses some precision and crispness.
First of all, thermal processing of aminoacids is a polycondensation process, as small molecules (water, ammonia) are side products (page 2).

We have added a paragraph to page 2 of our paper to clarify this point. The paragraph we added is as follows:
"Thermal proteins (proteinoids)~\cite{fox1992thermal} are synthesized by thermal polycondensation of amino acids. This involves heating a mixture of amino acids to 160-200~^{o}C under an inert atmosphere, triggering a polycondensation reaction between the amino acids. Rather than a typical polymerization which links monomer units together directly, this is a step-growth polymerization which also generates small molecule byproducts like water and ammonia. The high temperatures cause bifunctional amino acids like glutamic acid to cyclize, which facilitates their role as solvents and initiators for the polycondensation reaction. The end result is a complex mixture of polypeptides with a broad distribution of chain lengths~\cite{harada1958thermal,fox1992thermal}."

For the experimental part, detailed recipes, including amounts of reagents and detailed reaction conditions should be given.

We have revised the sentences to specify the amount of amino acids used:
"*The amino acids weighing 5 g in total were heated to their boiling points and mixed together in equimolar amounts.*"
"The resulting mixture was then dissolved in water at a temperature of 80 degrees Celsius, while continuously mixing, to achieve a concentration of 10 mg/100 ml for each protenoid."

For electrochemical measurement the configuration of the potentiostat should be given (2- or 3-electrode setup, concentration of protenoids, supporting electrolyte...). More detailed information of the experimental setup should be given: types of electrodes, materials of

electrodes, electrode spacing, etc. Detailed drawing of photo of the setup would be very beneficial.
Applied voltage values should be given for all experiments.

Here is a revised paragraph that has been expanded and improved for clarity and coherence:

‘’A two-electrode setup was used for the electrochemical measurements, with platinum and iridium-coated stainless steel sub-dermal needle electrodes (Spes Medica S.r.l., Italy) serving as the working and counter/reference electrodes. The electrodes were positioned approximately 10 mm apart in a protenoid solution containing 10 mg/100 ml protenoid in water as the supporting electrolyte. The potentiostat applied a constant dc potential (Edc) and measured the current response at 0.1 s intervals for 25,000 s. For the results shown, Edc was 0.01 V, with no initial equilibration period. The proteinoids exhibited varying spiking frequency and amplitude when exposed to different applied potentials, specifically positive and negative Edc values. Communication with the protenoids was established using this two-electrode electrochemical cell connected to the potentiostat.”

Authors present detailed analysis of signal statistical features, but use single current measurements for logic analysis - how the time-dependent variability of the signal (Figure 4 and 9) translate into logic performance. It may not be relevant, but should be briefly discussed, as the relevance of signal variability may potentially influence the performance ,especially when different protenoid will have significantly different time scale of variability.

We have added the following text in the discussion section:
‘’Although our analysis primarily focuses on using single current measurements for implementing logic gates, we acknowledge that the protenoid signals display time-dependent variability, as demonstrated in Figure~\ref{savdsbfgsndghmjmk,k}. Distinct time scales are observed for the spiking behaviour and noise characteristics of different proteonid types. The variability in the signals over time has the potential to affect the reliability and precision of logic operations, especially when using proteinoids with significantly different dynamics. Logic gating that relies on transient spikes may be impacted if the timing of the spikes is inconsistent. In order to obtain consistent logic inputs from protenoid currents that have a high degree of variability, it may be necessary to employ suitable signal processing or filtering techniques. Further investigation is needed to understand the impact of signal noise and fluctuations on logic accuracy. The present study showcases the use of simple DC signal levels to perform proof-of-concept logic operations. This serves as a starting point for further investigation into more intricate signal processing methods that can help reduce time-dependent variations.”
